# P-1389. Portrayal of Sexually Transmitted Infections in Popular Films

**DOI:** 10.1093/ofid/ofae631.1565

**Published:** 2025-01-29

**Authors:** Michael A Deaney, Kinsey McClure, Meghan N Jeffres

**Affiliations:** Denver Health Medical Center, Denver, Colorado; Huntsville Hospital, Huntsville, Alabama; University of Colorado Anschutz Medical Campus, Aurora, CO

## Abstract

**Background:**

Stigmatization of sexually transmitted infections (STIs) threatens provider-patient relationships and leads to the underutilization of STI services. Media can shape public attitudes toward healthcare topics by perpetuating or reducing stigma. Considering the prevalence of STIs and the widespread consumption of media, healthcare providers must be cognizant of factors influencing societal perceptions of STIs. This study aims to analyze the representation and accuracy of STIs in popular narrative films.

**Figure:**
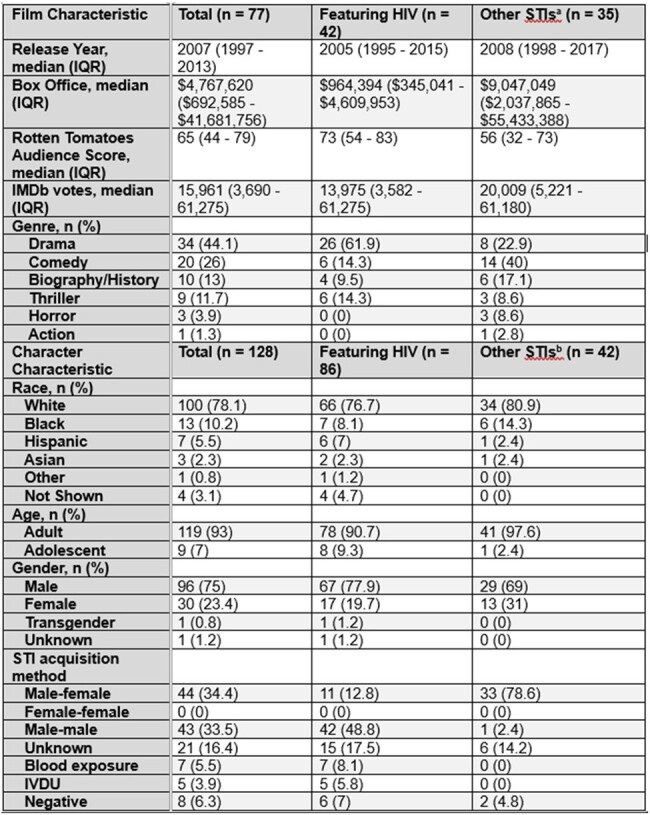

Characteristics of Films and Characters Portraying Sexually Transmitted Infections.

a Other STIs featured in films include HSV (n=11), gonorrhea (n=6), syphilis (n=8), pubic lice (n=4), unspecified STI (n=3), chlamydia (n=2), HPV (n=1), trichomoniasis (n=0).

b Other STIs featured in characters include HSV (n=13), gonorrhea (n=8), syphilis (n=8), pubic lice (n=5), unspecified STI (n=4), chlamydia (n=3), HPV (n=1), trichomoniasis (n=0).

**Methods:**

This study is a descriptive analysis of popular films depicting STIs. Full-length, English-language, narrative films featuring characters with confirmed or suspected STIs were gathered through a keyword search from the Internet Movie Database. Descriptive statistics were used to determine the proportion of characters with accurately portrayed STI clinical variables of symptoms, transmission, diagnosis, and treatment.

**Figure:**
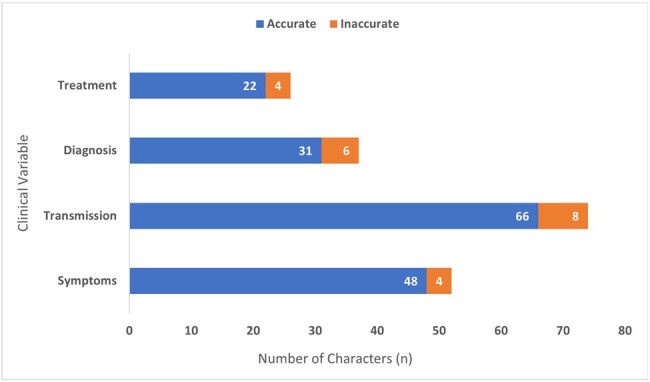

Stacked bar graph displaying proportion of accurate (blue) and inaccurate (orange) STIs in individual characters by clinical variable (symptoms, transmission, diagnosis, and treatment). Character counts are presented as data labels within their respective colors.

**Results:**

This study examined 128 characters from 77 films. Human immunodeficiency virus (HIV) was the most commonly portrayed STI in 42 films (55%). HIV was most commonly featured in dramas (62%). Herpes simplex virus, gonorrhea, syphilis, pubic lice, chlamydia, and human papillomavirus were most frequently portrayed in comedies (40%). Other characteristics are displayed in Table 1. Characters with STIs were primarily adult (93%), non-Hispanic White (78%), and male (75%). Figure 1 shows the rates of clinical variable depiction and accuracy by character. Accuracy of each clinical variable was 92% for symptoms, 89% for transmission, 84% for diagnosis, and 85% for treatment. Four (3%) characters represented all clinical variables, 2 (50%) of which were accurately portrayed. Death occurred in 39 characters (30%), with HIV contributing to the majority of deaths (82%).

**Conclusion:**

Films commonly represent accurate symptoms, transmission, diagnosis, and treatment of STIs. However, the frequency of death secondary to STIs is exaggerated and may contribute to STI stigmatization. Understanding the portrayal of STIs in popular films can provide valuable insights for clinicians, enabling them to address patient misconceptions and knowledge gaps.

**Disclosures:**

**All Authors**: No reported disclosures

